# Long-term Trajectories of Low Back Pain in Older Men: A Prospective Cohort Study With 10-Year Analysis of the Osteoporotic Fractures in Men Study

**DOI:** 10.1093/gerona/glae175

**Published:** 2024-07-12

**Authors:** David T McNaughton, Eric J Roseen, Sheena Patel, Aron Downie, Cecilie K Øverås, Casper Nim, Steen Harsted, Hazel Jenkins, James J Young, Jan Hartvigsen, Jessica J Wong, Katie L Stone, Kristine E Ensrud, Soomi Lee, Peggy M Cawthon, Howard A Fink

**Affiliations:** College of Health Sciences, School of Medical, Health, and Applied Sciences, Central Queensland University, Brisbane, Queensland, Australia; School of Psychological Sciences, Macquarie University, Sydney, New South Wales, Australia; Section of General Internal Medicine, Department of Medicine, Boston University Chobanian and Avedision School of Medicine, Boston Medical Center, Boston, Massachusetts, USA; Department of Physical Medicine and Rehabilitation, VA Boston Healthcare System, Boston, Massachusetts, USA; Research Institute, California Pacific Medical Center, San Francisco, California, USA; Department of Chiropractic, Macquarie University, Sydney, New South Wales, Australia; Department of Public Health and Nursing, Norwegian University of Science and Technology (NTNU), Trondheim, Norway; Spine Centre of Southern Denmark, University Hospital of Southern Denmark, Middelfart, Denmark; Department of Sports Science and Clinical Biomechanics, Center for Muscle and Joint Health, University of Southern Denmark, Odense, Denmark; Spine Centre of Southern Denmark, University Hospital of Southern Denmark, Middelfart, Denmark; Department of Sports Science and Clinical Biomechanics, Center for Muscle and Joint Health, University of Southern Denmark, Odense, Denmark; Department of Chiropractic, Macquarie University, Sydney, New South Wales, Australia; Department of Sports Science and Clinical Biomechanics, Center for Muscle and Joint Health, University of Southern Denmark, Odense, Denmark; Schroeder Arthritis Institute, Krembil Research Institute, University Health Network, Toronto, Canada; Department of Sports Science and Clinical Biomechanics, Center for Muscle and Joint Health, University of Southern Denmark, Odense, Denmark; Chiropractic Knowledge Hub, Odense, Denmark; Institute for Disability and Rehabilitation Research, Ontario Tech University, Oshawa, Canada; Research Institute, California Pacific Medical Center, San Francisco, California, USA; Department of Epidemiology and Biostatistics, University of California, San Francisco, San Francisco, California, USA; Division of Epidemiology and Community Health, Department of Medicine, University of Minnesota, Minneapolis, Minnesota, USA; Department of Human Development and Family Studies, Center for Healthy Aging, Pennsylvania State University, University Park, Pennsylvania, USA; Research Institute, California Pacific Medical Center, San Francisco, California, USA; Department of Epidemiology and Biostatistics, University of California, San Francisco, San Francisco, California, USA; Division of Epidemiology and Community Health, Department of Medicine, University of Minnesota, Minneapolis, Minnesota, USA; Center for Care Delivery and Outcomes Research, Minneapolis VA Health Care System, Minneapolis, Minnesota, USA

**Keywords:** Epidemiology, Latent growth curve analyses, Low back pain, Older adults

## Abstract

Although low back pain (LBP) may persist or recur over time, few studies have evaluated the individual course of LBP over a long-term period, particularly among older adults. Based on data from the longitudinal Osteoporotic Fractures in Men (MrOS) Study, we aimed to identify and describe different LBP trajectories in older men and characterize members in each trajectory group. A total of 5 976 community-dwelling men (mean age = 74.2) enrolled at 6 U.S. sites were analyzed. Participants self-reported LBP (yes/no) every 4 months for a maximum of 10 years. Latent class growth modeling was performed to identify unique LBP trajectory groups that explained variation in the LBP data. The association of baseline characteristics with trajectory group membership was assessed using univariable and multivariable multinominal logistic regression. A 5-class solution was chosen; *no/rare LBP* (*n* = 2 442/40.9%), *low frequency-stable LBP* (*n* = 1 040/17.4%), *low frequency-increasing* LBP (*n* = 719/12%), *moderate frequency-decreasing LBP* (*n* = 745/12.5%), and *high frequency-stable LBP* (*n* = 1 030/17.2%). History of falls (OR = 1.52), history of LBP (OR = 6.37), higher physical impairment (OR = 1.51–2.85), and worse psychological function (OR = 1.41–1.62) at baseline were all associated with worse LBP trajectory groups in this sample of older men. These findings present an opportunity for targeted interventions and/or management to older men with worse or increasing LBP trajectories and associated modifiable risk factors to reduce the impact of LBP and improve quality of life.

Low back pain (LBP) is a leading cause of disability (1) and healthcare and social support expenditures worldwide (2). Furthermore, it is anticipated that the burden of LBP on society will increase in the coming decades as the global average age is expected to increase (3). Although LBP is regularly categorized by the duration of a single episode (acute or chronic) (4), many people experience multiple episodes over years described as recurrent episodic, fluctuating, or continuous persistent pain (5). Consequently, a long-term perspective is imperative to identify distinct LBP trajectories within individuals and consider heterogeneity of the course of LBP within populations. The increasing burden globally of LBP among older adults emphasizes the need to improve the characterization of LBP trajectories across the life course, particularly into late life (6–8).

A prior study of LBP trajectories in 675 older adults (41% male and >55 years age) presenting with new LBP to their general practitioner and reassessed at up to 7 time points over a 3-year period observed 3 trajectories of back pain (low, moderate, or high levels of LBP) (9). Although this study, and others, inform the LBP trajectory literature, several limitations are common. These include small sample sizes (10), relatively short follow-up periods (typically up to 1 year) (11,12), a long time intervals between LBP assessments (eg, 2–5 years) (13,14). All these characteristics may increase the risk of misclassification of membership to pain trajectory groups over the long term. This is particularly relevant in trajectory research that investigates pain over the lifespan, which often include a limited proportion of older adults (>65 years) (13).

Additionally, most LBP trajectory studies have used care-seeking populations (5,9,11,15–18) limiting our understanding of the range of LBP experienced in non-care-seeking populations (19,20). Older adults with an increased risk for having worse trajectories and poor long-term outcomes may need better help to manage their LBP. However, the identification of modifiable risk factors for unfavorable pain trajectories is also understudied in older adults. By including only those seeking care for LBP (and excluding individuals without LBP) identification of characteristics that are associated with worse outcomes among an older non-care-seeking population may assist clinicians in targeting the use of evidence-based LBP treatment and prevention strategies.

The aim of this study is to uncover unique long-term LBP trajectories in community-dwelling older men (65+ years) over a 10-year period, and for each trajectory to explore baseline clinical, health, and sociodemographic characteristics that are associated with group membership.

## Method

We used data from the Osteoporotic Fractures in Men (MrOS) Study (https://mrosonline.ucsf.edu), a prospective multicenter longitudinal cohort study. Population and recruitment details have been previously described (21,22). Institutional review boards at all centers approved the study protocol and procedures were conducted in accordance with the ethical standards for human subjects’ research described in the Helsinki Declaration. These analyses followed reporting guidelines specified by Strengthening the Reporting of Observational Studies in Epidemiology (STROBE) (23).

### Study Population

A total of 5 994 community-dwelling men aged 65 years and older were enrolled in MrOS at 6 U.S. sites from March 2000 to April 2002: Birmingham, AL; Minneapolis, MN; Palo Alto, CA; Monongahela Valley near Pittsburgh, PA; Portland, OR; and San Diego, CA. The inclusion criteria were: (a) ability to walk without the assistance of another person, (b) absence of bilateral hip replacements, (c) ability to provide self-reported data, (d) anticipated residence near a clinical site for the duration of the study, and (e) absence of a medical condition that (in the judgment of the investigator) would result in imminent death. The original study design was to prospectively: (a) determine risk factors for osteoporosis and related fractures, (b) understand age-related medical conditions and risk factors, not limited to fall risk, and (c) understand the relationship with prostate disease in men 65 years of age and older. At the baseline visit, each participant was asked to provide written informed consent, complete the self-administered questionnaire, attend the clinic visit, and complete anthropometric measures.

### Measurements

#### Low back pain

Following the baseline visit, participants were asked to complete mailed questionnaires on LBP every 4 months. Participants were asked “have you experienced LBP in the previous 4 months” with a yes/no response. This item was the most relevant, widely used in previous studies on pain (24), and relatively easy to answer for the older participants. Up to 39 mailed questionnaires per participant were available for analysis utilizing this question.

#### Measure of time: age

Participant age (not rounded to whole numbers) was calculated based on the participant’s birthdate and the first day of the month each triannual questionnaire was mailed: March 1, July 1, and November 1.

#### Baseline characteristics

At baseline, self-reported demographic characteristics included race/ethnicity, educational attainment, marital status, and living arrangement as well as lifestyle habits (current alcohol consumption and smoking status). Medical history included self-reported physician diagnosis of the following: cardiovascular disease (stroke, myocardial infarction, or angina), cancer (nonskin), chronic obstructive pulmonary disease (COPD), diabetes, and arthritis. Self-reported medication use was also assessed for use of inhaled or oral corticosteroids and use of central nervous system agents including benzodiazepines, opioid analgesics, or antidepressants, and a total medication count.

Physical activity was measured using the Physical Activity Scale for the Elderly (PASE) (25), a 12-item questionnaire assessing activity level for leisure, household, and occupational activities. Impairment in instrumental activities of daily living (IADLs) was assessed based on self-reported difficulty preparing meals, doing heavy housework, or shopping for groceries or clothing. Impairment in physical function was defined based on self-reported difficulty walking 2 to 3 blocks on level ground or climbing 10 steps. Physical health and mental health were measured using the 12-item Short-Form (SF-12) physical component summary score and mental component summary score, respectively (26). Cognitive function was measured using the Trail-Making Task B (27) and Modified Mini-Mental Status (3MS) Examination (28). Depressive symptoms were measured using a question from the SF-12: “How much of the time during the past four weeks have you felt downhearted and blue?” Responses were classified as none of the time versus at least a little of the time. Fall history at baseline was based on self-report and classified as having no falls or at least one fall in the last 12 months. Fracture history at baseline was based on self-reported fracture since 50 years of age.

In-clinic physical measures (>90% of total sample completed at baseline) included weight, height, and total percent body fat by DXA. Body mass index (BMI) was calculated as weight (kg)/height (meters^2^). Walking speed (m/s) was measured using the timed completion of a 6-m course performed at the participant’s usual walking speed without any acceleration or deceleration (22). Number of chair stands per 10 seconds was calculated using the timed completion of 5 chair stands (29). Narrow walk time was measured using the timed completion of a 6-m course while taking no more than 2 deviations outside a narrow path (20 cm). Leg power (watts) was measured using the Nottingham Power Rig (Nottingham University, Nottingham, England). Grip strength (kg) was measured using Jamar dynamometers (Sammons Preston Rolyan, Bolingbrook, IL) (30).

### Statistical Approach

The analysis assumed that several distinct and unrecognized trajectories of LBP are revealed by patterns of recorded LBP frequency (yes/no) data across up to 39 time points over 10 years. Latent class growth analysis (LCGA) was used to identify such unique trajectory groups that explained heterogeneity in the data. Each participant was assigned to one of the identified trajectory groups based on highest probability of belonging. We then performed univariable and multivariable multinominal logistic regression analyses to assess the association of baseline characteristics with membership in each LBP trajectory class compared to no/rare LBP.

#### Group-based trajectory modeling

The *TRAJ* procedure (SAS Institute, Cary, NC) was used to identify unique groups of participants based on similar patterns of LBP frequency over time (pain phenotypes) (31,32). A data-driven approach for optimal model selection was conducted per literature guidance (33). That is, LCGA was conducted using a binary logistic distribution with a range of linear, quadratic, and cubic growth curves specified for up to a 6-class model. To determine the optimal form and number of classes in the model, we considered several goodness of fit indices, including the change in Bayesian Information Criteria (BIC), Akaike’s Information Criteria (AIC), Lo–Mendell–Rubin adjusted Likelihood Ratio Test (LMR-LRT) as well as the relative size of membership for each class as a proportion of the entire sample. Model adequacy was assessed based on the average posterior probabilities and relative entropy of membership placement in each trajectory class. Eighteen (0.3%) participants who had at least one missing value for the LBP variable from the mailed questionnaire during the first 2 years of follow-up were excluded from the analyses. LCGA uses maximum likelihood to assign participants to a trajectory class, so missing values (death, termination of participation, or missing responses after the first 2 years of the study period) are handled without need for imputation. Finally, the clinical interpretability of candidate models was considered. Once the final model was selected, characteristics of participants in each trajectory group were described.

#### Multinominal logistic regression analysis

Univariable and multivariable multinominal logistic regression analyses were used to compare baseline characteristics of participants in each trajectory group to the group with no/rare LBP using proportional classification for all participants (34). Associations were reported as odds ratios (OR) with 95% confidence intervals (CI). Continuous baseline variables were expressed as odds per standard deviation increase. Nonnormally distributed count or continuous variables were categorized in the following way: total medications trichotomized as 0, 1–3, or 4 plus medications; Teng 3MS was dichotomized at 80 points, with scores <80 reflecting cognitive impairment (28); and both SF-12 mental and physical summary scores were split at their median.

First, individual baseline characteristics were investigated, and then a single model was conducted with all baseline characteristics included. This approach initially resulted in 1 388 participants being excluded from the analyses due to missing data from the predictor variables (which was predominately due to 2 variables; Nottingham leg power and narrow walk speed). A decision to remove Nottingham leg power and narrow walk speed from the multivariable model was made to maximize model power. This approach led to only 606 (10%) participants with variables missing. All analyses were performed in SAS version 9.4 (SAS Institute), while a subset of graphs was generated using Stata v17 (17.1, StataCorp LLC, College Station, TX). A .05 significance level was used.

#### Post hoc sensitivity analyses

We conducted a post hoc sensitivity analysis to investigate the potential impact of missing data on final LCGA model. To do this, the LCGA was replicated in a truncated sample involving 20 mail questionnaires (omitting 19 mailed questionnaires), thus including fewer missing data but over a shorter period of time. The proportion of participants in the 5-class model, model fit statistics, and observation of trajectory shape were then compared to the initial 5-class LCGA presented in text.

## Results

**Figure 1.**
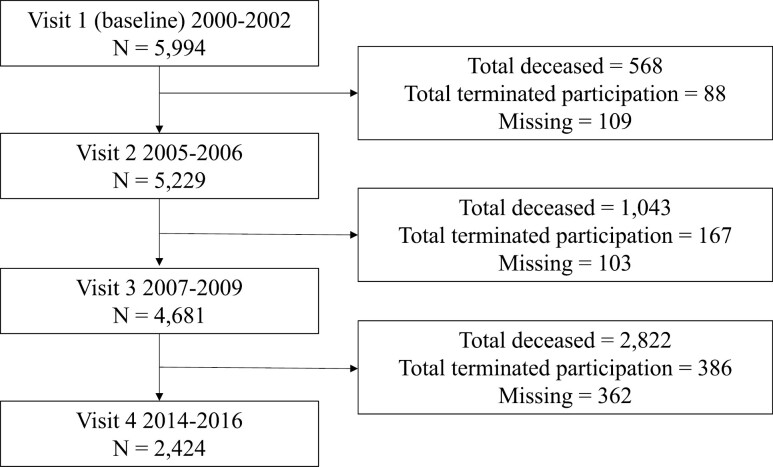
Participant flow up to Study Visit 4 (2014–2016). Mailed questionnaires (up to 39 responses) utilized for the LCGA analysis were completed up to 9.75 years after 2000–2002 (between Visit 3 and Visit 4). At the time of the 39th mailed questionnaire, *N* = 2 684 men had either died or terminated participation.

**Figure 2.**
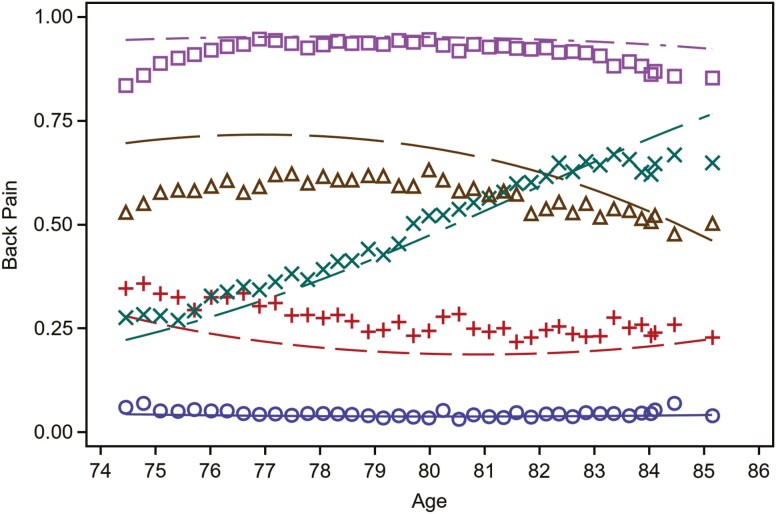
LBP trajectories by age (5-class solution). Note: Class 1(**○**): *no/rare LBP* (*N* = 2 442/40.9%), 2(+): *low frequency-stable LBP* (*N* = 1 040/17.4%), 3(×): *low frequency-increasing LBP* (*N* = 719/12%), 4(Δ): *moderate frequency-decreasing LBP* (745/12.5%) and 5(□): *high frequency-stable LBP* (*N* = 1 030/17.2%). Interpretation of trajectory classes: Class 1 = low probability (<10%) of reporting LBP at each assessment; Class 2 = ~25% probability of reporting LBP at each assessment; Class 3 = increasing probability of reporting LBP at each assessment from ~25% to ~60%; Class 4 = between 50% and 75% probability of reporting LBP at each assessment; Class 5: high probability (>75%) of reporting LBP at each assessment.

The analytic sample included 5 976 men (mean age = 74.14 [*SD* = 5.88]; White race *n* = 5 346 [90%]). Figure 1 illustrates participant dropout up to Visit 4 (2014–2016), and as of the final mailed questionnaire utilized in the current study (maximum 39 responses/9.75 years after study enrollment), 2 684 men had either died or terminated participation. Based off the LCGA, 5 trajectory classes were identified as *no/rare LBP* (*N* = 2 442/40.9%; reference class), *low frequency-stable LBP* (*N* = 1 040/17.4%), *low frequency-increasing* LBP (*N* = 719/12%), *moderate frequency-decreasing LBP* (*N* = 745/12.5%), and *high frequency-stable LBP* (*N* = 1 030/17.2%; Figure 2).

### Selection of Ideal Number of Trajectories

Table 1 displays goodness of fit criteria, average posterior probability and proportion of participants classified up to a 6-class model. Supplementary Figure 1a–f illustrate the 1–6 class models. A 5-class quadratic model was chosen based on changes in BIC, AIC, and LMR-LRT, and balancing model complexity against interpretability (Table 1). Other models with either linear or cubic fit (Supplementary Table 1a and b) did not meaningfully improve model fit or uncover additional meaningful sub-groups beyond the chosen model. Mean posterior probabilities for the chosen model were acceptable (0.85–0.93), relative entropy was 0.85 and the membership of the smallest class was 12% (*N* = 719), leaving acceptable power for all subsequent analyses.

**Table 1.** Goodness of Fit Criteria, Model Adequacy and Proportional Placement for 1-Class to 6-Class Models

Goodness of Fit Criteria									Average Posterior Probability						Relative Entropy	Percent in Each Class					
Class	Order	*n*	LL	LMR-LR	LMR-LR P	AIC	BIC	Δ BIC	Class 1	Class 2	Class 3	Class 4	Class 5	Class 6		Class 1	Class 2	Class 3	Class 4	Class 5	Class 6
1	Linear	5 976	−114 754.4	—	—	−114 756.4	−114 766.48	—	100.00	—	—	—	—	—	—	100.00	—	—	—	—	—
2	Quadratic	5 976	−79 992.71	66 956.056	*p* < .001	−79 999.708	−80 035.013	34 731.5	0.99	0.98	—	—	—	—	0.96	63.52	36.48	—	—	—	—
3	Quadratic	5 976	−74 435.97	10 703.13	*p* < .001	−74 446.97	−74 502.449	5 532.6	0.97	0.94	0.97	—	—	—	0.92	50.57	26.94	22.49	—	—	—
4	Quadratic	5 976	−72 938.16	2 885.012	*p* < .001	−72 953.155	−73 028.808	1 473.6	0.94	0.88	0.91	0.95	—	—	0.87	39.90	24.55	18.48	17.07	—	—
**5**	**Quadratic**	**5** **976**	**−71** **613.98**	**2** **550.63**	***p* < .001**	**−71** **632.977**	−**71728.804**	**1** **300.0**	**0.85**	**0.93**	**0.85**	**0.87**	**0.93**	—	**0.85**	**17.97**	**39.63**	**12.78**	**12.85**	**16.77**	—
6	Quadratic	5 976	−70 777.17	1 611.816	*p* < .001	−70 800.167	−70 916.168	812.6	0.85	0.90	0.84	0.86	0.90	0.82	0.83	10.44	35.57	11.67	10.90	13.57	17.85

*Notes*: Change in the Bayesian Information Criterion (BIC) measures the weight of evidence against the previous, less complex model. The best-fit model is related to the smallest negative value. AIC = Akaike Information Criterion; BIC = Bayesian Information Criterion; ΔBIC: Change in BIC from the previous model; LL = Log-likelihood; LMR-LR = Lo-Mendell-Rubin likelihood ratio test statistic; LMR-LR P = *p*-value for the Lo-Mendell-Rubin test; n = Sample size.

Average posterior probability is a measure of the internal reliability for each trajectory class (values closer to 1.0 are ideal).

Entropy is the ability of the model to provide well-separated classes (closer to 1.0 is ideal). A value greater than 0.80 indicates less classification uncertainty.

Bold indicates chosen 5-class solution.

### Description of the Five-Class Trajectory Model

Figure 2 illustrates the chosen 5-class trajectory model. On average, participants in Class 1 *(no/rare LBP*, 40.9%) had a less than 10% probability of reporting LBP at each assessment; Class 2 (*low frequency-stable LBP*, 17.4%) had ~25% probability of reporting LBP at each assessment; Class 3 (*low frequency-increasing LBP*, 12%) the probability of reporting LBP increased from ~25% to ~60% over 10 years; Class 4 (*moderate frequency-decreasing LBP,* 12.5%) probability of reporting LBP decreased from ~60% to less than 50% over 10 years; and Class 5 (*high frequency-stable LBP*, 17.2%) had >75% probability of reporting LBP at each assessment.

Supplementary Figure 2a–e displays mailed questionnaire responses from a selection of participants to illustrate typical longitudinal LBP patterns within each trajectory class. Throughout the study period, the response rate to the mailed questionnaires for active participants was above 98%. Supplementary Figure 3 illustrates the negligible difference in response rate by participants across trajectory classes.

Baseline characteristics of the 5 trajectory classes are presented in Table 2. Several differences between trajectory classes were identified and include education level, chronic disease diagnosis (arthritis, COPD, or cardiovascular disease), analgesic medication use, mental and physical quality of life, and IADL/ADL limitations. There were many characteristics that do not seem to be helpful for predicting class membership and include living arrangement, marital status, diabetes or cancer diagnosis, fracture status since age 50, maximum grip strength, and cognitive impairment.

**Table 2.** Baseline Characteristics for Each Trajectory Group

	*n*	Overall Sample	No/Rare LBP	Low Frequency-Stable LBP	Low Frequency-Increasing LBP	Moderate Frequency-LBP	High Frequency-Stable LBP
		(*N* = 5 976)	(*N* = 2 442)	(*N* = 1 040)	(*N* = 719)	(*N* = 745)	(*N* = 1 030)
Age	5 976	74.14 ± 5.88	74.06 ± 5.99	74.52 ± 5.62	74.20 ± 5.93	73.72 ± 5.75	74.23 ± 5.90
White race	5 976	5 346 (89.46)	2 167 (88.74)	908 (87.31)	655 (91.10)	679 (91.14)	937 (90.97)
Body mass index	5 969	27.38 ± 3.83	27.06 ± 3.69	27.15 ± 3.67	27.75 ± 3.96	27.56 ± 3.91	28.00 ± 4.08
Education (high school+)	5 976	5 585 (93.46)	2 304 (94.35)	981 (94.33)	670 (93.18)	684 (91.81)	946 (91.84)
Depressive symptoms	5 975	296 (4.95)	102 (4.18)	61 (5.87)	37 (5.15)	39 (5.23)	57 (5.53)
Greater/equal to 1 alcoholic drink per week	5 968	3 113 (52.16)	1 298 (53.24)	547 (52.60)	398 (55.51)	384 (51.61)	486 (47.23)
Current smoker	5 975	206 (3.45)	74 (3.03)	39 (3.75)	23 (3.20)	31 (4.16)	39 (3.79)
Lives alone	5 976	835 (13.97)	318 (13.02)	149 (14.33)	113 (15.72)	108 (14.50)	147 (14.27)
Married	5 976	4 918 (82.30)	2 038 (83.46)	853 (82.02)	573 (79.69)	616 (82.68)	838 (81.36)
Arthritis (body) or gout	5 976	2 834 (47.42)	906 (37.10)	454 (43.65)	374 (52.02)	408 (54.77)	692 (67.18)
Arthritis (low back)	5 976	970 (16.23)	140 (5.73)	137 (13.17)	118 (16.41)	148 (19.87)	427 (41.46)
COPD	5 976	637 (10.66)	187 (7.66)	121 (11.63)	75 (10.43)	92 (12.35)	162 (15.73)
CVD (MI, stroke, or angina)	5 976	1 626 (27.21)	549 (22.48)	278 (26.73)	213 (29.62)	230 (30.87)	356 (34.56)
Diabetes	5 976	651 (10.89)	238 (9.75)	117 (11.25)	87 (12.10)	81 (10.87)	128 (12.43)
Cancer (nonskin)	5 976	1 088 (18.21)	426 (17.44)	200 (19.23)	130 (18.08)	130 (17.45)	202 (19.61)
Fall in last 12 months	5 976	1 262 (21.12)	403 (16.50)	240 (23.08)	153 (21.28)	179 (24.03)	287 (27.86)
Fracture since age 50	5 976	98 (1.64)	35 (1.43)	13 (1.25)	15 (2.09)	15 (2.01)	20 (1.94)
Antidepressant use	5 635	351 (6.23)	86 (3.76)	56 (5.79)	57 (8.28)	57 (8.05)	95 (9.68)
Benzodiazepine use	5 740	205 (3.57)	58 (2.48)	30 (3.04)	30 (4.30)	23 (3.20)	64 (6.42)
Opioid use	5 740	158 (2.75)	21 (0.90)	16 (1.62)	13 (1.87)	25 (3.48)	83 (8.32)
NSAID use	5 740	888 (15.47)	224 (9.57)	133 (13.48)	118 (16.93)	133 (18.52)	280 (28.08)
ADL limitation	5 976	830 (13.89)	185 (7.58)	120 (11.54)	110 (15.30)	129 (17.32)	286 (27.77)
IADL limitation (y/*n*)	5 976	845 (14.14)	186 (7.62)	116 (11.15)	107 (14.88)	138 (18.52)	298 (28.93)
Narrow walk-in m/s using best time	5 421	1.15 ± 0.27	1.16 ± 0.26	1.15 ± 0.26	1.15 ± 0.28	1.14 ± 0.27	1.10 ± 0.27
Walk speed in m/s using both times	5 964	1.20 ± 0.23	1.22 ± 0.22	1.21 ± 0.22	1.20 ± 0.22	1.20 ± 0.23	1.15 ± 0.24
PASE Score	5 973	146.50 ± 68.16	150.30 ± 69.58	145.04 ± 65.23	144.80 ± 69.06	146.53 ± 68.82	140.10 ± 66.03
Max of *L*/*R* grip strength, KG	5 862	41.63 ± 8.50	41.72 ± 8.33	41.54 ± 8.47	41.72 ± 8.57	41.50 ± 8.69	41.57 ± 8.75
Chair stands per 10 s (those unable/refused = 0)	5 953	4.73 ± 1.52	4.91 ± 1.47	4.72 ± 1.51	4.71 ± 1.44	4.63 ± 1.54	4.39 ± 1.61
Nottingham maximum power, both legs, watts	5 426	208.46 ± 63.08	209.58 ± 62.97	206.39 ± 64.36	212.93 ± 61.96	209.35 ± 63.45	203.83 ± 62.26
Total number of meds: (0, 1–3 or 4+)		672 (11.93)	344 (15.02)	125 (12.91)	73 (10.61)	74 (10.45)	56 (5.71)
	5 635	2 164 (38.40)	985 (43.01)	373 (38.53)	230 (33.43)	257 (36.30)	319 (32.52)
		2 799 (49.67)	961 (41.97)	470 (48.55)	385 (55.96)	377 (53.25)	606 (61.77)
Cognitive impairment trails B (>1.5 *SD* above mean)	5 835	541 (9.27)	215 (9.01)	84 (8.29)	66 (9.36)	75 (10.29)	101 (10.07)
Cognitive impairment, Teng 100 (<80)	5 972	167 (2.80)	66 (2.70)	33 (3.17)	12 (1.67)	26 (3.49)	30 (2.92)
SF-12 modified mental sum scale (median split)	5 973	2 985 (49.97)	1 079 (44.22)	556 (53.51)	386 (53.69)	388 (52.08)	576 (55.92)
SF-12 modified physical sum scale (median split)	5 973	2 976 (49.82)	864 (35.41)	502 (48.32)	377 (52.43)	466 (62.55)	767 (74.47)

*Notes*: ALD = activities of daily living; IADL = instrumental activities of daily living; COPD = chronic obstructive pulmonary disease; CVD = cardiovascular disease; LBP = low back pain; NSAID = non-steroidal anti-inflammatory.

### Univariable and Multivariable Multinominal Logistic Regression Analyses

Supplementary Table 2 displays the results from the univariable multinominal logistic regression analyses. Six hundred and six participants were excluded due to missing values related to predictor variables, leaving *N* = 5 370 for the multivariable multinominal logistic regression analyses.

**Figure 3.**
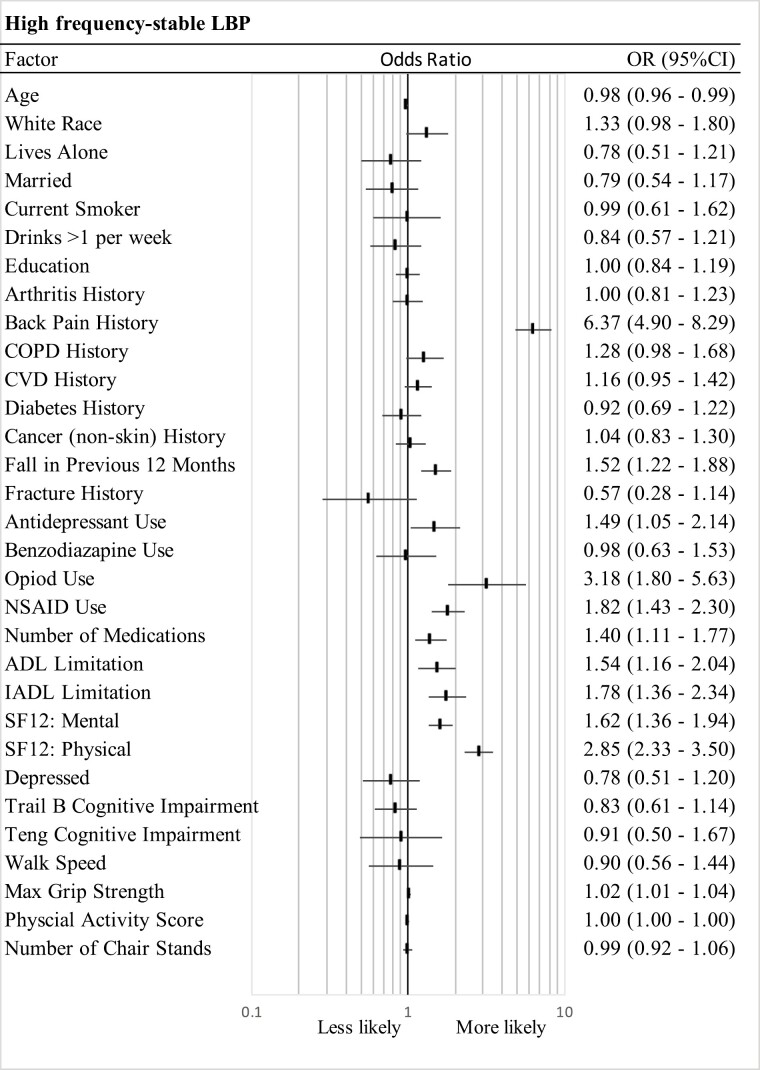
Multivariable multinominal logistic regression analysis. Odds ratios and 95% CI estimates for trajectory Class 5 (*high frequency-stable LBP*) compared to trajectory class 1 (*no/rare LBP*). ALD = activity of daily living; COPD = chronic obstructive pulmonary disease; CVD = cardiovascular disease; IADL = instrumental activities of daily living; NSAID = non-steroidal anti-inflammatory use.

Figure 3 and Supplementary Figure 4a–c illustrate the odds of belonging to each trajectory class compared to the reference class, *no/rare LBP* trajectory (class 1), based on the multivariable multinominal logistic regression analyses. Several baseline characteristics were consistently associated with worse LBP trajectories (ie, classes with high or increasing frequency of LBP), which included previous LBP, presence of medical comorbidities, higher likelihood of a fall history, increased likelihood of analgesic, anti-inflammatory and antidepressant medication use, higher likelihood of a worse IADL and ADL limitation, and worse mental and physical quality of life. The magnitude of these OR estimates appeared to increase with worsening trajectory of class membership.

#### Comparison high frequency-stable LBP (Class 5) with no/rare LBP (Class 1)

Relative to participants in the *no/rare LBP* class, those with *high frequency-stable LBP* were slightly younger (OR = 0.98 [0.96–0.99]), were more likely to have reported LBP on entry (OR = 6.37 [4.90–8.29]), and more likely to have fallen in the previous 12 months (OR = 1.52 [1.22–1.88]; Figure 3). These participants were also more likely to have reported antidepressant, opioid, NSAID, and total medication count use on entry (OR = 1.49 [1.05–2.14]; OR = 3.18 [1.80–5.63]; OR = 1.82 [1.43–2.30]; OR = 1.40 [1.11–1.77], respectively). Finally, this trajectory class had a greater likelihood of reporting a worse ADL and IADL limitation (OR = 1.54 [1.16–2.04]; OR = 1.78 [1.36–1.94], respectively) and was more likely to be categorized with a worse mental (OR = 1.62 [1.36–1.94]) and physical quality of life (OR = 2.85 [2.33–3.50]).

#### Comparison moderate frequency-decreasing LBP (Class 4) with no/rare LBP (Class 1)

Relative to participants in the *no/rare LBP* class, participants with *moderate frequency-decreasing LBP* were slightly younger (OR = 0.97 [0.95–0.97]) and more likely to be White race (OR = 1.46 [1.06–2.01]; Supplementary Figure 4a). These participants were less likely to report more than 1 alcoholic drink per week (OR = 0.59 [0.41–0.85]), had a greater likelihood of reporting LBP at baseline (OR = 2.57 [1.91–3.44]), falling in the past 12 months (OR = 1.38 [1.10–1.73]) and reporting opioid use (OR = 2.01 [1.05–3.86]) at baseline. Finally, this trajectory class had a greater likelihood of reporting a worse IADL limitation (OR = 1.51 [1.12–1.69]), and worse mental (OR = 1.41 [1.17–1.69]) and physical quality of life (OR = 2.47 [2.01–3.03]).

#### Comparison low frequency-increasing LBP (Class 3) with no/rare LBP (Class 1)

Relative to participants with *no/rare LBP* class, participants with *low frequency-increasing LBP* were more educated (OR = 1.23 [1.02–1.48]), had a greater likelihood of LBP before baseline (OR = 2.04 [1.50–2.77]), more often reported antidepressant use (OR = 1.60 [1.09–2.34]), NSAID use (OR = 1.42 [1.09–1.85]), and had been categorized with a worse mental and physical quality of life (OR = 1.51 [1.25–1.81]; OR = 1.59 [1.30–1.95], respectively; Supplementary Figure 4b).

#### Comparison low frequency-stable LBP (Class 2) with no/rare LBP (Class 1)

Relative to participants within the *no/rare LBP* class, participants with *low frequency-stable LBP* had a greater likelihood of reporting LBP prior to baseline (OR = 2.21 [1.65–2.95]) and COPD (OR = 1.36 [1.04–1.76]), were more likely to have fallen in the past 12 months (OR = 1.44 [1.18–1.77]), and had been categorized with a worse mental and physical quality of life (OR = 1.53 [1.30–1.80]; OR = 1.57 [1.31–1.88], respectively; Supplementary Figure 4c).

### Post Hoc Sensitivity Analysis

Supplementary Table 3 displays model fit statistics, average posterior probabilities, and relative entropy of the truncated model (first 20 mailed questionnaires). When compared to the full model (all 39 mailed questionnaires), the truncated model had a similar proportion of class membership across all classes, high average posterior probabilities indicating a high chance of class belonging and acceptable model fit statistics. Further, similar LBP trajectory descriptors were identified (Supplementary Figure 5), except for class 3 (*stable-increasing LBP*). In the truncated model, participants in class 3 did not increase their probability of reporting LBP over time, likely due to the (post hoc) truncated time period. This further supports use of all 39 questionnaires to uncover meaningful trajectory patterns in these data.

## Discussion

The current study described the 10-year trajectory of LBP among men aged 65 years and older. Five trajectory classes were identified with worse trajectory severity being associated with a range of potentially modifiable sociodemographic, medical, lifestyle, and functional characteristics. Our analyses characterized the course of LBP in this sample and a significant proportion of participants rarely reported LBP (40%), which did not worsen over time.

Low back pain trajectories have been identified across several populations and studies (5); however, there is a significant lack of research investigating older adults. This is important because older adults comprise a growing proportion of the population (3,7), and attention to the health problems of older people and to factors that may be modifiable are clinically important. Previously reported qualitative experiences of LBP in older adults align with our study findings, in that LBP affects a broad range of physical, psychological, and social factors (35). It is also important to note a large proportion of the sample (40%) was categorized in the *no/rare LBP* class. So, although LBP is common within those with advancing age to varying levels of frequency, a significant proportion of older men will experience LBP rarely and this does not necessarily indicate a progressive or worsening LBP disease status.

Previous trajectory analyses investigating LBP among older adults have drawn participants exclusively from care-seeking samples and not from the general population (9,15,16,36). There are obvious differences in measurement between these previous studies and our analyses, with all utilizing intensity and disability measures of LBP as an outcome measure. Further, the previous literature generally reflects short-term (<24 months) with high-frequency measurements (weekly or monthly) (9,15,16,36) or long-term (10–20 years) with low-frequency measurements (1–5 years) (13,14). Although there are strengths and limitations to both approaches when determining LBP disease burden, the key aspect to the current study is the balance between frequent measurement (every 4 months) and long follow-up duration (10 years). Despite these measurement differences, balanced measurement timing and reporting period, the trajectory classes identified in the present study are generally consistent reporting stable low, medium, and high LBP severity trajectories (9,15,16,36).

This study identified a trajectory where the probability of reporting LBP steadily increased (*low frequency-increasing LBP*), which is a novel finding given the 10-year study period and advanced age of this population. Further, characteristics of this group (*low frequency-increasing LBP*) mirror those seen in the more frequent and severe LBP reporting trajectory classes. As some of the characteristics are potentially modifiable (eg, ADL limitations, antidepressant use, and mental quality of life), this unfortunate trajectory may be preventable, and an important target for intervention. Specifically, older individuals utilizing antidepressants, functional impairment, and worse mental quality of life should be targeted for evidence-based LBP strategies even if LBP is infrequent, given the increased risk of increasing LBP episodes over time.

Our study reaffirms the importance of considering the previous history of an individual’s LBP when communicating prognosis to individuals with LBP. Put differently, those with a history of LBP on presentation are likely to continue to experience LBP into the future. This finding highlights the importance of identifying effective management strategies for those with existing LBP and implementing strategies to prevent the initial onset of LBP at younger ages. In addition, the improved identification of individuals likely to have worse long-term LBP trajectories based on baseline characteristics and short-term trajectories will facilitate design of potential intervention studies, including which populations to target for evaluation.

Several characteristics such as use of more medications and certain classes of medications, greater physical impairments and worse psychological functioning were consistently associated with worse LBP trajectory classes. In contrast, our measure of depressive symptoms was not significantly associated with worse LBP trajectories. This may be more related to a limitation of the measure available, especially in the context of previous research that has identified a relationship between depression and worse LBP trajectories in older adults (37). This suggests early and tailored management/interventions to physical impairments and psychological functioning may be targeted, using a range of physical and psychological interventions (such as cognitive behavioral therapy), among older men with low, moderate, and high frequency LBP reporting (38).

Finally, an unfortunate reality of longitudinal data in a population of older adults is the potential impact of death or dropout over the course of the study period. There were several aspects to our analytical approach that mitigated against the effects of survivor bias. Firstly, we used all available data, as selecting only participants with complete data across the study period would increase the level of true survivor bias in our results. Second, LCGA utilizes maximum likelihood to assign participants to a trajectory class; therefore, missingness is handled without the need for imputation. Third, we found high posterior probability (membership) for each class, which makes it unlikely that class membership would change based on death or dropout over the study period. Finally, we conducted a post hoc LCGA sensitivity analysis by modeling 5-classes using only the first 20 mailed questionnaires (instead of all 39 mailed questionnaires). Compared to the full model, the truncated model returned similar model fit statistics, similar (high) average posterior probabilities, and similar trajectories.

### Strengths and Limitations

There were several strengths to the current study. Firstly, this was based on a large sample of older men with frequent LBP measurements over a 10-year period. Second, the response rate for active participants to the mailed questionnaires was above 98%. Finally, the sample reflects a non-care-seeking population, which is more likely reflective of LBP disease estimates in the community. There were, however, several limitations that should be addressed. Importantly, the study sample predominantly reflects older White community-dwelling men, and therefore generalizability to other populations may be limited. In addition, an inclusion criterion for the current study was absence of medical conditions, such as metastatic cancer, which would result in imminent death. This potentially resulted in a sample of more healthy individuals, which limits our understanding of other populations who have more comorbid and serious chronic disease. However, retention of the cohort over time was extremely high among survivors and thus, the healthy volunteer selection bias was attenuated by long-term follow-up. Missing data due to dropouts may have introduced attrition bias; however, we conducted a post hoc sensitivity analysis, which suggests that attrition unlikely had a meaningful impact on the interpretation of the presented LCGA.

We also had limited information prior to enrollment in the study; therefore, the temporal effect of many characteristics on the development and trajectory of LBP prior to study enrollment is unknown. For example, it is unknown whether opioid use preceded LBP reporting or was a consequence of it. The LBP outcome used (binary yes/no) cannot be directly compared to studies that measured trajectory of LBP intensity or disability scores. Despite these differences, we did observe similar low, medium, and high trajectory patterns to those previously reported (5). There was no information regarding treatment for LBP over the study period, which may modify trajectory classification and/or the observed associations. Several of the measures utilized are self-reported, including physical activity scales. Additionally, although we included psychological variables that broadly captured psychological health, other measures that are specific to pain such as pain catastrophizing or kinesiophobia may be more predictive of worsening or severe pain trajectories. Finally, our measurement timing was restricted to a 4-month recall period; while this is superior to 12- or 24-month recall periods common in other long-term follow-up studies, it may still introduce recall and misclassification bias (24).

### Future Directions

Future research should replicate these findings in women, non-White and non-U.S. populations. Further, the use of objective and pain-specific measures would provide a more detailed description of worse LBP trajectory classes. Finally, subsequent analyses examining the association between long-term LBP trajectories with the risk of clinical outcomes and healthcare utilization would provide important information for the appropriate distribution of healthcare resources and targeting of clinical treatments.

## Conclusion

Approximately 60% of older men frequently reported LBP over a 10-year period. In contrast, 40% of individuals infrequently reported LBP. Importantly, we identified a low frequency-increasing LBP trajectory class, reflecting an increased reporting of LBP, and this pattern has not been observed in prior studies of LBP trajectories in older adults. Comorbid health conditions, history of falls, history of LBP, higher medication use, greater physical impairment, and worse psychological function at baseline were all associated with worse LBP trajectory classes in this sample of older men. Many of these associated modifiable risk factors may be targets for early, tailored, and individualized evidence-based management.

## Supplementary Material

glae175_suppl_Supplementary_Material

## Data Availability

The data that support the findings of this study are openly available at https://mrosonline.ucsf.edu.
