# Supplementary material for: Long-term Trajectories of Low Back Pain in Older Men: A Prospective Cohort Study With 10-Year Analysis of the Osteoporotic Fractures in Men Study
Source: J Gerontol A Biol Sci Med Sci. 2024 Jul 12;79(9):glae175. doi: 10.1093/gerona/glae175 (PMC11333921; doi:10.1093/gerona/glae175)
Supplement: glae175_suppl_Supplementary_Material [file glae175_suppl_supplementary_material.docx]

**Supplementary Material**

| **METHOD: ALL LINEAR** | | | | | | | | |
| --- | --- | --- | --- | --- | --- | --- | --- | --- |
| **Classes** | **Order** | **n** | **LL** | **LMR-**  **LR** | **LMR-**  **LR P** | **AIC** | **BIC** | **Δ BIC** |
| 1 | linear | 5976 | -114754.4 |  |  | -114756.40 | -114766.48 |  |
| 2 | linear | 5976 | -80032.79 | 66878.817 | p < 0.001 | -80037.79 | -80063.01 | 34703.5 |
| 3 | linear | 5976 | -74519.48 | 10619.535 | p < 0.001 | -74527.48 | -74567.83 | 5495.2 |
| 4 | linear | 5976 | -73023.57 | 2881.353 | p < 0.001 | -73034.57 | -73090.05 | 1477.8 |
| 5 | linear | 5976 | -72541.22 | 929.183 | p < 0.001 | -72555.22 | -72625.83 | 464.2 |
| 6 | linear | 5976 | -71582.93 | 1845.845 | p < 0.001 | -71599.93 | -71685.67 | 940.2 |
| **METHOD: ALL QUADRATIC** | | | | | | | | |
| **Classes** | **Order** | **n** | **LL** | **LMR- LR** | **LMR- LR P** | **AIC** | **BIC** | **Δ BIC** |
| 1 | quadratic | 5976 | -114753.6 |  |  | -114756.64 | -114771.77 |  |
| 2 | quadratic | 5976 | -79992.71 | 66956.056 | p < 0.001 | -79999.708 | -80035.013 | 34736.8 |
| 3 | quadratic | 5976 | -74435.97 | 10703.13 | p < 0.001 | -74446.97 | -74502.449 | 5532.6 |
| 4 | quadratic | 5976 | -72938.16 | 2885.012 | p < 0.001 | -72953.155 | -73028.808 | 1473.6 |
| **5** | **quadratic** | **5976** | **-71613.98** | **2550.63** | **p < 0.001** | **-71632.977** | **-71728.804** | **1300.0** |
| 6 | quadratic | 5976 | -70777.17 | 1611.816 | p < 0.001 | -70800.167 | -70916.168 | 812.6 |
| **METHOD: ALL CUBIC** | | | | | | | | |
| **Classes** | **Order** | **n** | **LL** | **LMR- LR** | **LMR- LR P** | **AIC** | **BIC** | **Δ BIC** |
| 1 | cubic | 5976 | -114746.2 |  |  | -114750.16 | -114770.33 |  |
| 2 | cubic | 5976 | -79985.03 | 66955.479 | p < 0.001 | -79994.03 | -80039.42 | 34730.9 |
| 3 | cubic | 5976 | -74411.07 | 10736.26 | p < 0.001 | -74425.07 | -74495.68 | 5543.7 |
| 4 | cubic | 5976 | -72918.69 | 2874.611 | p < 0.001 | -72937.69 | -73033.51 | 1462.2 |
| 5 | cubic | 5976 | -71791.21 | 2171.753 | p < 0.001 | -71815.21 | -71936.25 | 1097.3 |
| 6 | cubic | 5976 | -70551.74 | 2387.484 | p < 0.001 | -70580.74 | -70727.00 | 1209.3 |

Table S1a. Trajectory model goodness of fit criteria with alternative form

| **Linear** | | | | | | | | | | | | | | |
| --- | --- | --- | --- | --- | --- | --- | --- | --- | --- | --- | --- | --- | --- | --- |
|  | **Average posterior probability** | | | | | | **Relative**  **Entropy** | **Percent in each class** | | | | | | **Order** |
| **n** | **Class**  **1** | **Class**  **2** | **Class**  **3** | **Class**  **4** | **Class**  **5** | **Class**  **6** |  | **Class**  **1** | **Class**  **2** | **Class**  **3** | **Class**  **4** | **Class**  **5** | **Class**  **6** | **significance** |
| 5976 | 100.00 |  |  |  |  |  |  | 100.00 |  |  |  |  |  | significant |
| 5976 | 0.99 | 0.98 |  |  |  |  | 0.96 | 63.52 | 36.48 |  |  |  |  | Class 1 not significant |
| 5976 | 0.97 | 0.94 | 0.97 |  |  |  | 0.92 | 50.74 | 27.019 | 22.241 |  |  |  | Class 1 not significant |
| 5976 | 0.95 | 0.89 | 0.91 | 0.95 |  |  | 0.87 | 39.8603 | 24.73 | 18.60 | 16.81 |  |  | Class 1 not significant |
| 5976 | 0.85 | 0.90 | 0.86 | 0.87 | 0.91 |  | 0.82 | 23.9649 | 33.50 | 16.65 | 13.17 | 12.71 |  | Class 1,2 not significant |
| 5976 | 0.81 | 0.81 | 0.83 | 0.85 | 0.89 | 0.89 | 0.81 | 18.38 | 12.93 | 10.63 | 10.56 | 13.19 | 34.30 | all significant |
| **Quadratic** | | | | | | | | | | | | | | |
|  | **Average posterior probability** | | | | | | **Relative**  **Entropy** | **Percent in each class** | | | | | | **Order** |
| **n** | **Class**  **1** | **Class**  **2** | **Class**  **3** | **Class**  **4** | **Class**  **5** | **Class**  **6** |  | **Class**  **1** | **Class**  **2** | **Class**  **3** | **Class**  **4** | **Class**  **5** | **Class**  **6** | **significance** |
| 5976 | 100.00 |  |  |  |  |  |  | 100.00 |  |  |  |  |  | not significant |
| 5976 | 0.99 | 0.98 |  |  |  |  | 0.96 | 63.52 | 36.48 |  |  |  |  | all significant |
| 5976 | 0.97 | 0.94 | 0.97 |  |  |  | 0.92 | 50.57 | 26.94 | 22.49 |  |  |  | all significant |
| 5976 | 0.95 | 0.88 | 0.91 | 0.95 |  |  | 0.87 | 39.90 | 24.55 | 18.48 | 17.07 |  |  | all significant |
| **5976** | **0.85** | **0.93** | **0.85** | **0.87** | **0.93** |  | **0.85** | **17.97** | **39.63** | **12.78** | **12.85** | **16.77** |  | **all significant** |
| 5976 | 0.85 | 0.91 | 0.84 | 0.86 | 0.90 | 0.82 | 0.83 | 10.44 | 35.57 | 11.67 | 10.90 | 13.57 | 17.85 | all significant |
| **Cubic** | | | | | | | | | | | | | | |
|  | **Average posterior probability** | | | | | | **Relative**  **Entropy** | **Percent in each class** | | | | | | **Order** |
| **n** | **Class**  **1** | **Class**  **2** | **Class**  **3** | **Class**  **4** | **Class**  **5** | **Class**  **6** |  | **Class**  **1** | **Class**  **2** | **Class**  **3** | **Class**  **4** | **Class**  **5** | **Class**  **6** | **significant** |
| 5976 | 100.00 |  |  |  |  |  |  | 100.00 |  |  |  |  |  | all significant |
| 5976 | 0.99 | 0.98 |  |  |  |  | 0.96 | 63.56 | 36.44 |  |  |  |  | all significant |
| 5976 | 0.97 | 0.94 | 0.97 |  |  |  | 0.92 | 50.54 | 27.00 | 22.45 |  |  |  | Class 1,3,4 not significant |
| 5976 | 0.94 | 0.88 | 0.91 | 0.95 |  |  | 0.87 | 39.94 | 24.45 | 18.38 | 17.23 |  |  | all significant |
| 5976 | 0.84 | 0.93 | 0.85 | 0.86 | 0.93 |  | 0.84 | 20.51 | 38.72 | 11.36 | 12.69 | 16.72 |  | Class 5 not significant |
| 5976 | 0.83 | 0.85 | 0.85 | 0.85 | 0.91 | 0.91 | 0.83 | 14.93 | 13.74 | 11.04 | 9.63 | 13.76 | 36.91 | Class 5 not significant |

Table S1b.

Trajectory class membership and model adequacy with alternative form

Table S2.

Unadjusted multinominal logistic regression analyses for each trajectory class compared to trajectory class 1 (No LBP).

|  | **n** | **No/rare LBP** | **Low frequency - Stable**  **LBP** | **Low frequency - Increasing**  **LBP** | **Moderate frequency**  **LBP** | **High frequency - Stable**  **LBP** |
| --- | --- | --- | --- | --- | --- | --- |
| Age | 5976 | (ref) | 1.07 (1.00 - 1.14) | 1.02 (0.95-1.09) | 0.95 (0.89-1.02) | 1.02 (0.96-1.09) |
| White Race | 5976 | (ref) | 0.87 (0.70-1.09) | 1.30 (0.98- 1.728) | 1.31 (0.99-1.73) | 1.28 (0.99-1.64) |
| Body Mass Index | 5969 | (ref) | 1.02 (0.95 -1.10) | 1.2 (1.11-1.30) | 1.14 (1.05-1.24) | 1.27 (1.18-1.37) |
| Education (High school+) | 5976 | (ref) | 0.99 (0.73-1.36) | 0.82 (0.59-1.15) | 0.67 (0.49-0.92) | 0.68 (0.51-0.89) |
| Depressed | 5975 | (ref) | 1.43 (1.03 - 1.99) | 1.24 (0.85 - 1.83) | 1.27 (0.87- 1.85) | 1.34 (0.96- 1.87) |
| Greater/equal to 1 alcoholic drink per week | 5968 | (ref) | 0.97 (0.84-1.13) | 1.10 (0.93-1.30) | 0.94 (0.80-1.10) | 0.79 (0.68-0.91) |
| Current smoker | 5975 | (ref) | 1.25 (0.84-1.85) | 1.06 (0.66-1.70) | 1.39 (0.91-2.13) | 1.26 (0.85-1.87) |
| Lives alone | 5976 | (ref) | 1.12 (0.91-1.38) | 1.25 (0.99-1.57) | 1.13 (0.90-1.43) | 1.11 (0.90-1.37) |
| Married | 5976 | (ref) | 0.90 (0.75-1.09) | 0.78 (0.63-0.96) | 0.95 (0.76-1.18) | 0.87 (0.72-1.05) |
| Arthritis (body) or gout | 5976 | (ref) | 1.31 (1.13-1.52) | 1.84 (1.55-2.17) | 2.05 (1.74-2.42) | 3.47 (2.98-4.05) |
| Arthritis (Low back) | 5976 | (ref) | 2.49 (1.95-3.19) | 3.22 (2.49-4.19) | 4.07 (3.18-5.22) | 11.63 (9.42-14.37) |
| COPD | 5976 | (ref) | 1.59 (1.24-2.02) | 1.40 (1.06-1.86) | 1.70 (1.30-2.21) | 2.25 (1.80-2.82) |
| CVD (MI, stroke, or angina) | 5976 | (ref) | 1.26 (1.07-1.49) | 1.45 (1.21-1.75) | 1.54 (1.28-1.85) | 1.82 (1.55-2.14) |
| Diabetes | 5976 | (ref) | 1.17 (0.93-1.48) | 1.28 (0.98-1.66) | 1.13 (0.87-1.48) | 1.31 (1.05-1.65) |
| Cancer (non-skin) | 5976 | (ref) | 1.13 (0.94-1.36) | 1.05 (0.84-1.30) | 1.00 (0.81-1.24) | 1.16 (0.96-1.39) |
| Fall in last 12 months | 5976 | (ref) | 1.52 (1.27-1.82) | 1.37 (1.11-1.68) | 1.60 (1.31-1.95) | 1.96 (1.64-2.32) |
| Fracture since age 50 | 5976 | (ref) | 0.87 (0.46-1.65) | 1.47 (0.80-2.70) | 1.41 (0.77-2.60) | 1.36 (0.78-2.37) |
| Antidepressant use | 5635 | (ref) | 1.57 (1.11-2.22) | 2.32 (1.64-3.27) | 2.24 (1.59-3.17) | 2.75 (2.03-3.72) |
| Benzodiazepine use | 5740 | (ref) | 1.23 (0.79-1.93) | 1.77 (1.13-2.77) | 1.30 (0.80-2.13) | 2.70 (1.88-3.88) |
| Opioid use | 5740 | (ref) | 1.82 (0.95-3.50) | 2.1 (1.05-4.22) | 3.99 (2.22-7.16) | 10.03 (6.18-16.29) |
| NSAID use | 5740 | (ref) | 1.472 (1.17-1.85) | 1.93 (1.51-2.45) | 2.15 (1.70-2.71) | 3.69 (3.04-4.49) |
| ADL limitation | 5976 | (ref) | 1.59 (1.25-2.02) | 2.20 (1.71-2.84) | 2.56 (2.01-3.25) | 4.69 (3.83-5.74) |
| IADL limitation | 5976 | (ref) | 1.52 (1.19-1.94) | 2.12 (1.65-2.73) | 2.76 (2.17-3.50) | 4.94 (4.04-6.04) |
| Narrow walk-in m/s using best time | 5421 | (ref) | 0.93 (0.87-1.09) | 0.96 (0.88-1.05) | 0.92 (0.85-1.01) | 0.80 (0.74-0.86) |
| Walk speed in m/s using both times | 5964 | (ref) | 0.94 (0.88-1.02) | 0.91 (0.83-0.99) | 0.90 (0.83-0.98) | 0.72 (0.67-0.79) |
| PASE Score | 5973 | (ref) | 0.93 (0.86-0.99) | 0.92 (0.85-1.00) | 0.95 (0.87-1.03) | 0.86 (0.80-0.93) |
| Max of L/R grip strength, KG | 5862 | (ref) | 0.98 (0.91-1.05) | 1.00 (0.92-1.09) | 0.98 (0.90-1.06) | 0.98 (0.91-1.09) |
| Chair stands per 10 seconds (those unable / refused = 0) | 5953 | (ref) | 0.88 (0.81-0.94) | 0.87 (0.80-0.95) | 0.83 (0.76-0.90) | 0.71 (0.66-0.76) |
| Nottingham Maximum power, both legs, watts | 5426 | (ref) | 0.95 (0.88-1.03) | 1.05 (0.97-1.15) | 0.99 (0.92-1.09) | 0.91 (0.84-0.99) |
| Medication use | 5635 | (ref) | 1.24 (1.04-1.47) | 1.52 (1.24-1.86) | 1.45 (1.19-1.77) | 2.49 (2.04-3.03) |
| Cognitive Impairment Trails B (>1.5 SD above mean) | 5835 | (ref) | 0.91 (0.70-1.19) | 1.04 (0.78-1.39) | 1.16 (0.88-1.53) | 1.13 (0.88-1.45) |
| Cognitive Impairment, Teng 100 (<80) | 5972 | (ref) | 1.18 (0.77-1.80) | 0.61 (0.33-1.14) | 1.30 (0.82-2.07) | 1.08 (0.70-1.67) |
| SF12 modified mental sum scale (median split) | 5973 | (ref) | 1.45 (1.26-1.68) | 1.46 (1.24-1.73) | 1.37 (1.16-1.62) | 1.6 (1.38-1.85) |
| SF12 modified physical sum scale (median split) | 5973 | (ref) | 1.71 (1.47-1.98) | 2.01 (1.70 - 2.38) | 3.05 (2.57-3.61) | 5.32 (4.52-6.26) |

Results presented as Odds Ratios and 95% confidence intervals.

ADL = Activities of daily living. IADL = Instrumental Activities of Daily Living. NSAID = non-steroidal anti-inflammatory drugs. COPD = Chronic Obstructive Pulmonary Disease. CVD = cardiovascular disease

Table S3.

Goodness of fit criteria, model adequacy and proportional placement for 1-class to 6-class models using truncated sample (mailed questionnaires 1-20).

| **Goodness of fit criteria** | | | | | | | **Average posterior probability** | | | | | | **Relative**  **Entropy** | **Percent in each class** | | | | | | |
| --- | --- | --- | --- | --- | --- | --- | --- | --- | --- | --- | --- | --- | --- | --- | --- | --- | --- | --- | --- | --- |
| **Class** | **Order** | **n** | **LL** | **AIC** | **BIC** | **Δ BIC** | **Class**  **1** | **Class**  **2** | **Class**  **3** | **Class**  **4** | **Class**  **5** | **Class**  **6** |  | **Class**  **1** | **Class**  **2** | **Class**  **3** | **Class**  **4** | **Class**  **5** | **Class**  **6** |  |
| 1 | linear | 5976 | -71026.416 | -71028.416 | -71038.026 |  | 100.00 | - | - | - | - | - | - | 100.00 | - | - | - | - | - |  |
| 2 | quadratic | 5976 | -49301.083 | -49308.083 | -49341.717 | 21696.3 | 0.99 | 0.98 | - | - | - | - | 0.95 | 65.15 | 34.85 | - | - | - | - |  |
| 3 | quadratic | 5976 | -46254.127 | -46265.127 | -46317.980 | 3023.7 | 0.96 | 0.93 | 0.96 | - | - | - | 0.90 | 49.83 | 27.40 | 22.77 | - | - | - |  |
| 4 | quadratic | 5976 | -45553.299 | -45568.299 | -45640.371 | 677.6 | 0.95 | 0.85 | 0.89 | 0.92 | - | - | 0.84 | 42.63 | 24.56 | 17.08 | 15.73 | - | - |  |
| **5** | **quadratic** | **5976** | **-45408.488** | **-45427.488** | **-45518.779** | **121.6** | **0.82** | **0.89** | **0.80** | **0.82** | **0.86** | **-** | **0.78** | **22.73** | **38.42** | **14.65** | **12.70** | **11.49** | **-** |  |
| 6 | quadratic | 5976 | -44967.184 | -44990.18 | -45100.695 | 418.1 | 0.77 | 0.88 | 0.77 | 0.80 | 0.89 | 0.75 | 0.76 | 11.86 | 35.73 | 9.37 | 9.27 | 14.27 | 19.50 |  |


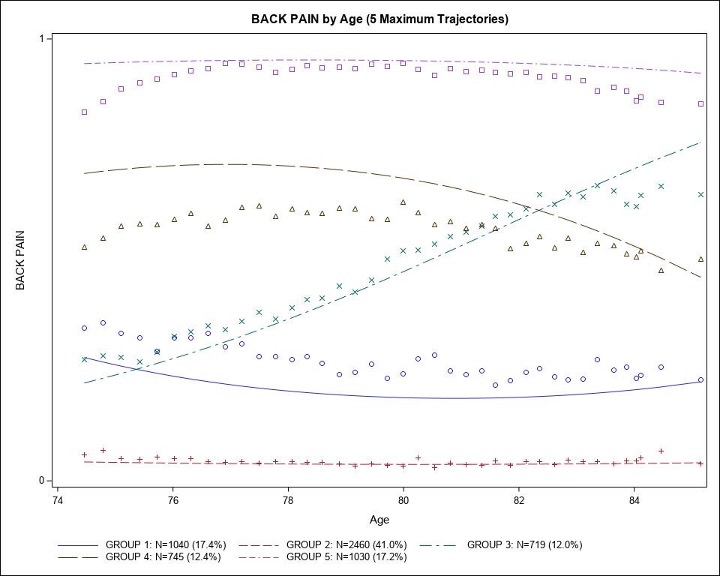

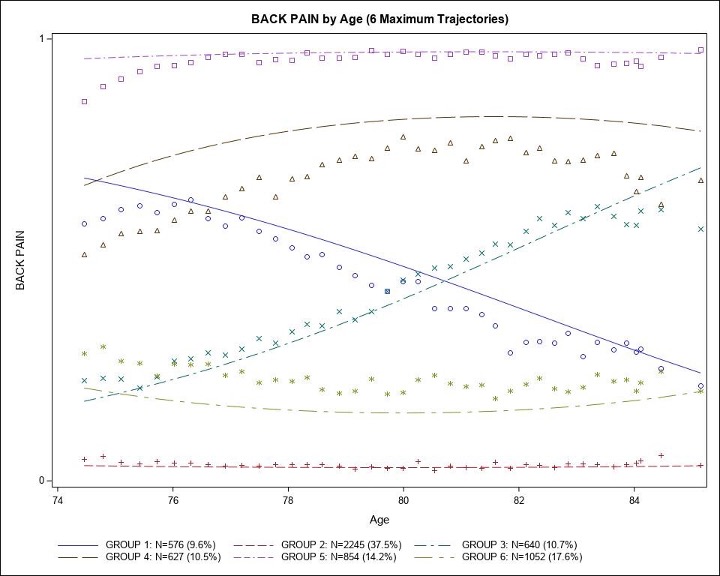

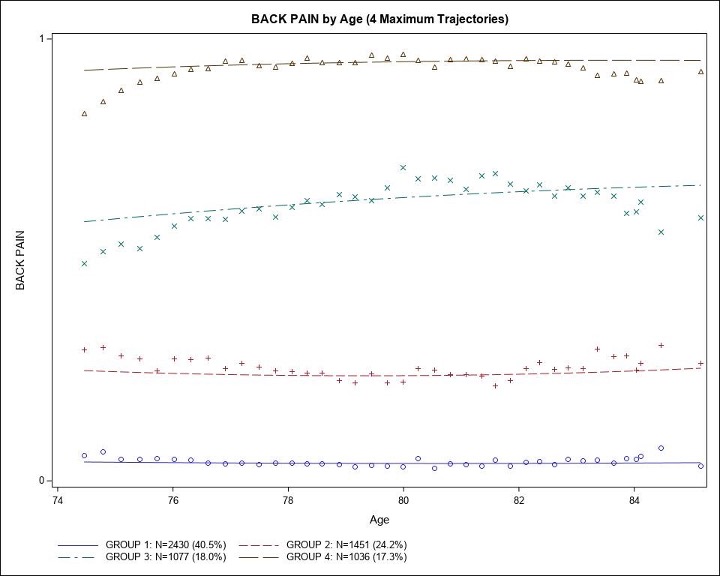

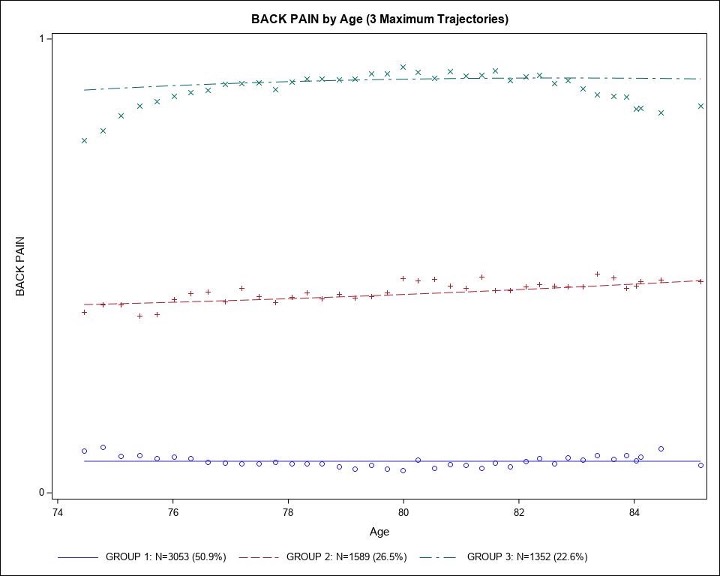

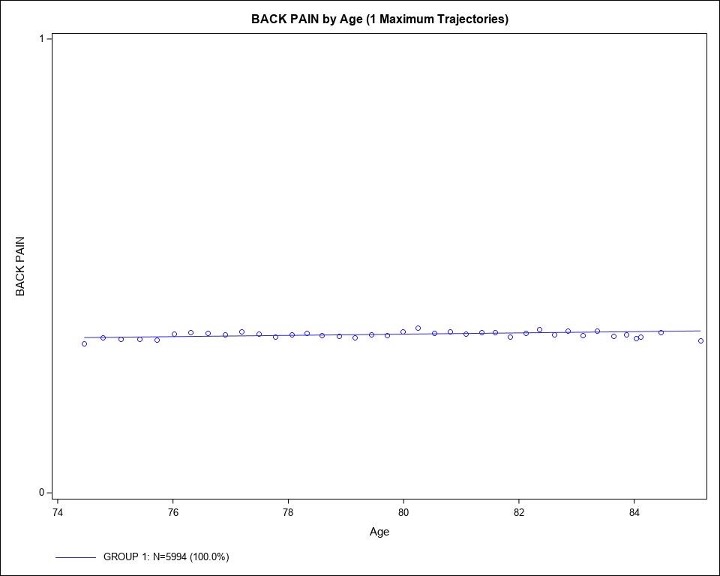

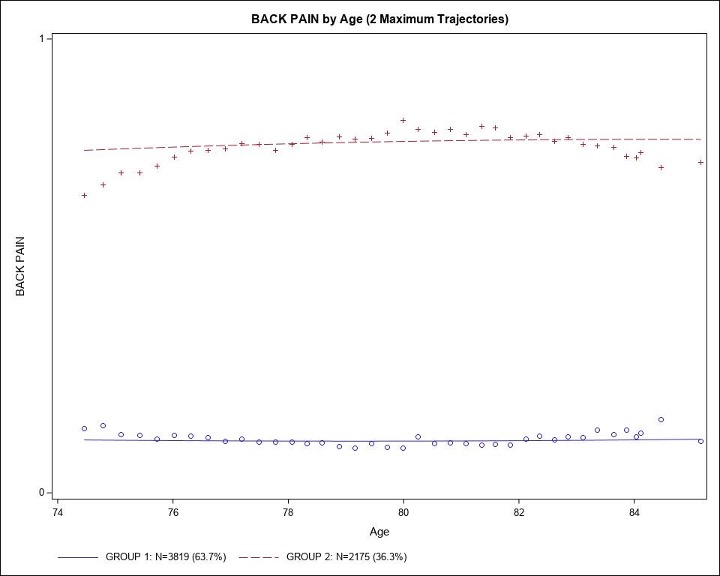


**Fig. S1c**

**Fig. S1f**

**Fig. S1e**

**Fig. S1d**

**Fig. S1a**

**Fig. S1b**

Figure S1a-f. Class trajectory options from 1-class solution to 6-class solutions.

Figure S2a. Individual mail questionnaire responses by a subset of participants from trajectory class 1 (*No/rare LBP)*. 0 = no LBP reported in previous 4 months, 1 = LBP reported in previous 4 months.

Figure S2b. Individual mail questionnaire responses by a subset of participants from trajectory class 2 (*Low frequency-stable LBP)*. 0 = no LBP reported in previous 4 months, 1 = LBP reported in previous 4 months.

Figure S2c. Individual mail questionnaire responses by a subset of participants from trajectory class 3 (*Low frequency-increasing LBP)*. 0 = no LBP reported in previous 4 months, 1 = LBP reported in previous 4 months.

Figure S2d. Individual mail questionnaire responses by a subset of participants from trajectory class 4 (*moderate frequency-decreasing LBP)*. 0 = no LBP reported in previous 4 months, 1 = LBP reported in previous 4 months.

Figure S2e. Individual mail questionnaire responses by a subset of participants from trajectory class 5 (*high frequency-stable LBP)*. 0 = no LBP reported in previous 4 months, 1 = LBP reported in previous 4 months.


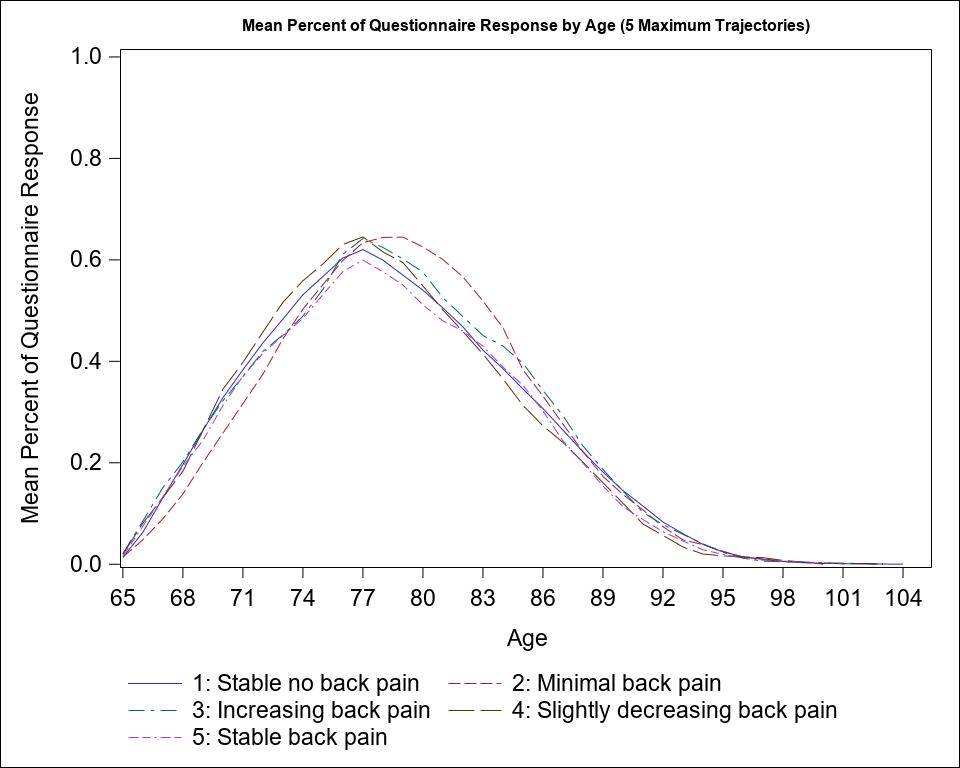


Figure S3 – Mean proportion of responses to mailed questionnaires by trajectory classes (final 5-class solution).

Denominator was total analytic sample (N=5976), which includes participant dropouts and those who died over time. If inclusion was limited to active participants only, mailed questionnaire response rate was above 98%.

Mean age at enrollment was 74 years.

Interpretation: Negligible differences in response rate between observed trajectory classes.

Multivariable Multinominal Logistic Regression Analysis.


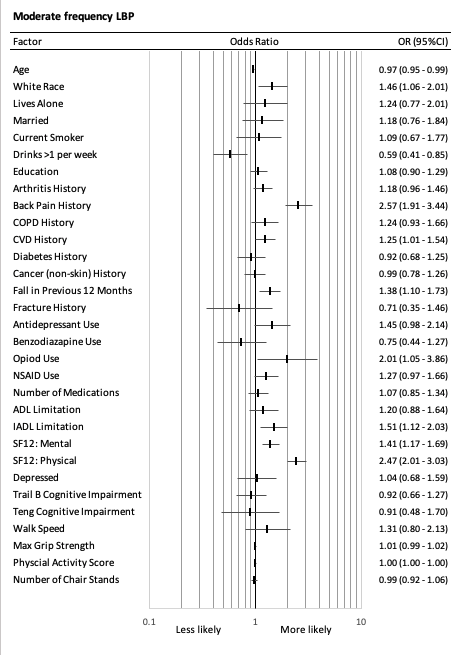


**Moderate frequency-decreasing LBP**

Odds ratios and 95% CI estimates for trajectory class 2 (*Moderate frequency-decreasing LBP)* compared to trajectory class 1 (*No/rare LBP*).


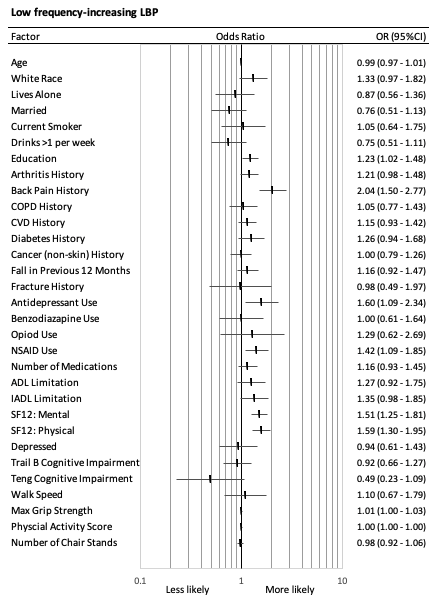


**Low frequency-increasing LBP**

Figure S4b.

Multivariable Multinominal Logistic Regression Analysis.

Odds ratios and 95% CI estimates for trajectory class 2 (*Low frequency-increasing LBP*) compared to trajectory class 1 (*No/rare LBP*).


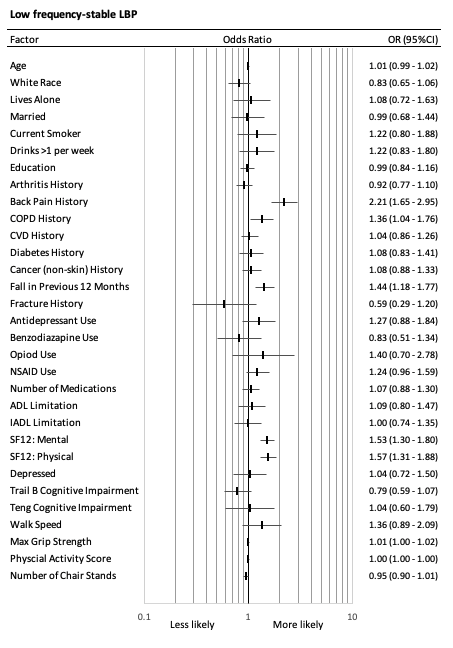


**Low frequency-stable LBP**

Figure S4c. Multivariable Multinominal Logistic Regression Analysis.

Odds ratios and 95% CI estimates for trajectory class 2 (*Low frequency-stable LBP*) compared to trajectory class 1 *(No/rare LBP)*


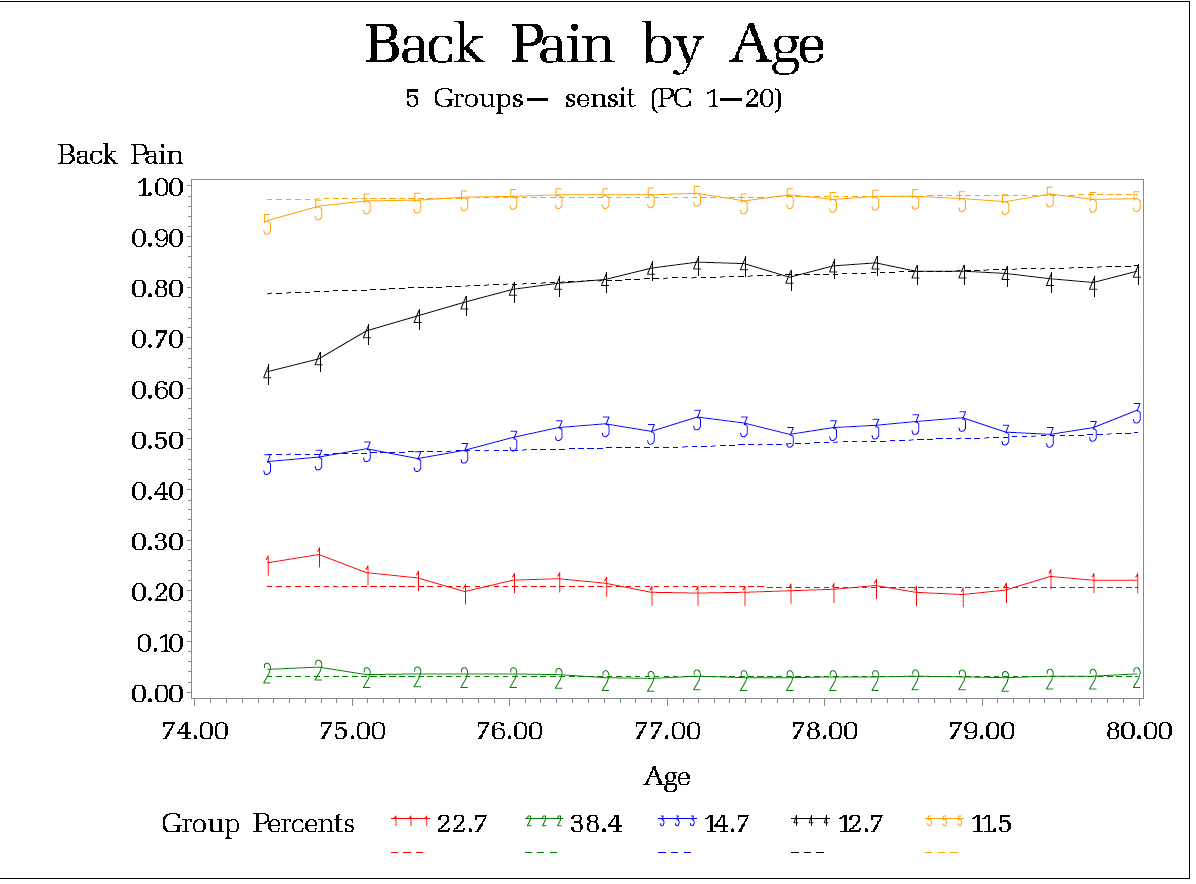


Figure S5. Sensitivity LCGA using truncated sample of 20 mailed questionnaires.
